# Intradialytic versus home based exercise training in hemodialysis patients: a randomised controlled trial

**DOI:** 10.1186/1471-2369-10-2

**Published:** 2009-01-29

**Authors:** Kirsten P Koh, Robert G Fassett, James E Sharman, Jeff S Coombes, Andrew D Williams

**Affiliations:** 1 School of Human Life Sciences, University of Tasmania, Newnham, Tasmania 7250, Australia; 2 School of Human Movement Studies, The University of Queensland, St Lucia, Queensland 4072, Australia; 3 Renal Research Tasmania, Launceston General Hospital, Charles St Launceston, Tasmania 7005, Australia; 4 Renal Medicine, Royal Brisbane and Women's Hospital, Brisbane, Queensland 4029, Australia; 5 School of Medicine, University of Queensland, Brisbane, Queensland 4029, Australia

## Abstract

**Background:**

Exercise training in hemodialysis patients improves fitness, physical function, quality of life and markers of cardiovascular disease such as arterial stiffness. The majority of trials investigating this area have used supervised exercise training during dialysis (intradialytic), which may not be feasible for some renal units. The aim of this trial is to compare the effects of supervised intradialytic with unsupervised home-based exercise training on physical function and arterial stiffness.

**Methods and design:**

This is a randomised, controlled clinical trial. A total of 72 hemodialysis patients will be randomised to receive either six months of intradialytic exercise training, home-based exercise training or usual care. Intradialytic patients will undergo three training sessions per week on a cycle ergometer and home-based patients will be provided with a walking program to achieve the same weekly physical activity. Primary outcome measures are six-minute walk distance (6 MWD) and aortic pulse wave velocity (PWV). Secondary outcome measures include augmentation index, peripheral and central blood pressures, physical activity and self-reported health. Measures will be made at baseline, three and six months.

**Discussion:**

The results of this study will help determine the efficacy of home-based exercise training in hemodialysis patients. This may assist in developing exercise guidelines specific for these patients.

**Trial Registration:**

ACTRN12608000247370

## Background

A number of studies have shown that intradialytic exercise training has positive effects on patients, such as improving cardiorespiratory fitness, physical function and self-reported health [1]. Less work has focussed on the effects of exercise training on cardiovascular risk factors in dialysis patients although some evidence suggests improvements in fasting glucose and insulin, an enhancement in the management of hypertension, and a reduction in inflammation [2-5].

Cardiovascular pathologies observed in ESKD patients include left ventricular hypertrophy and arterial disease [6]. In addition to the characteristic lesions of atherosclerosis, dialysis patients also experience thickening and fibrosis of the arterial wall in response to pressure and volume overload, loss of elastic fibres and medial fibrosis [7]. Such arterial remodelling, along with medial calcification, causes arteries to stiffen, thereby exacerbating left ventricular dysfunction [8]. Indeed, widening of the pulse pressure, the simplest and crudest method of assessing arterial stiffness, has proven to be a better marker of mortality than systolic pressure in haemodialysis patients [9]. Stiffening also results in more rapid propagation of the arterial pulse pressure wave through the conductance arteries and can be readily assessed by measuring aortic pulse wave velocity (PWV; a marker of regional large artery stiffness) and central augmentation index (AIx; a composite marker of systemic arterial stiffness and left ventricular systolic loading). Indeed, there is a clear evidence base that measures of arterial stiffness are independent predictors of cardiovascular morbidity and mortality in different patient populations [10] but particularly in ESKD patients [11-13].

To date, to our knowledge, only two studies have investigated the effect of exercise training on arterial stiffness in ESKD [14,15]. In an uncontrolled study of 11 hemodialysis patients, Mustata and coworkers [14] found a significant improvement in AIx after three months of supervised exercise training. More recently, aortic PWV was significantly decreased following three months of intradialytic exercise in 19 hemodialysis patients [15].

The majority of studies investigating the effects of exercise in dialysis patients have used supervised intradialytic exercise training. These programs are resource intensive which may preclude their use in clinical practice. Consequently other interventions should be investigated. To our knowledge only one study has compared different training regimens in an attempt to optimize the benefits of exercise training in ESKD patients [16]. Therefore, the aims of this study are to compare the effects of supervised intradialytic or unsupervised home-based exercise training on arterial stiffness and physical function in hemodialysis patients.

## Methods

### Study design and setting

The study is a multi-centre, randomized controlled trial in hemodialysis patients. The study will be conducted at the Launceston General Hospital and Burnie Satellite Renal Units in Northern Tasmania and the Hobart Renal Unit, which, combined, service a population of approximately 485,000.

### Ethical considerations

The Tasmanian Statewide Scientific and Ethics Committees have approved the trial. The Ethics Committee will be provided with annual reports of the trial progress and will promptly receive all adverse event reports. A condition of ethical approval requires investigators to provide brochures to all hemodialysis patients at each of the sites indicating the benefits of exercise in end stage kidney disease. Hence, potential benefits of regular exercise will be made known to patients regardless of their decision to participate in the study or the group to which they are randomised.

### Identification of eligible patients

Patients who commence and/or maintain dialysis for at least three-months will be approached to participate in this study. Eligible patients will receive a copy of the patient information sheet placed in the medical record. The principal investigator will then explain the study during a clinical consultation. After the explanation the subjects will be provided with a patient information sheet and informed consent form. The subject will then be asked to take this away with them and make a decision before their next hemodialysis visit. If the subject agrees to participate, they will sign the consent form with an independent person signing as a witness.

### Eligibility

Inclusion criteria are; age >18 and < 85 years incident and/or prevalent dialysis patients. Subjects will be excluded if they have unstable angina, lower limb amputation, or if they already meet or exceed the exercise recommendation of 120 minutes of moderate intensity physical activity per week[17] Subjects will also be excluded if they are participating in, or propose to participate in, another clinical intervention study within 30 days prior to study entry.

### Randomization

An individual not associated with the trial will perform the randomization using a computer generated random number system.

### Interventions

Intradialytic training requires participants to train on cycle ergometers (Rehab Trainer 881E, Monark, Sweden) within the first two hours of each dialysis session, three times per week for 6 months. Intensity will be individualised on the basis of perceived exertion, exercise heart rate and blood pressure. Participants will be requested to exercise at an RPE of 12–13 on the Borg 6–20 scale [18]. In cases where patients' training heart rates are low at a reported RPE of 12–13, the supervisor will increase the resistance to elicit a greater cardiorespiratory response. However, patients will be permitted to stop and rest or request to train at a lower intensity. In the event that exercise blood pressure exceeds predetermined safe levels for the participant, (>200/110 mmHg) they will be instructed to temporarily cease exercise and will be monitored until blood pressure returns to safe levels. Patients on medications that affect cardiac sinus rhythm will be trained strictly according to reported RPE. Over the duration of the exercise intervention, supervisors will periodically increase the resistance of the ergometer to maintain RPE. While participants will be encouraged to start and progress the duration of exercise according to their individual capabilities, a general guideline used by investigators will be for participants to complete fifteen minutes of exercise per session in the first two weeks of the intervention, progress to thirty minutes of exercise per session by week 12 and to forty-five minutes by week 24. Power output (w) and duration (minutes) of each exercise session will be recorded to estimate participants' individual energy expenditure per session during the training period.

Home-based participants will be asked to perform thrice weekly unsupervised walking for six months at perceived exertions of 12–13 on the Borg 6–20 scale. To ensure a similar treatment to the intradialytic group's training duration, home-based participants will be requested to start and progress their walking program according to individual capabilities however investigators will encourage participants to start at fifteen minutes per session for the first two weeks and to progress to 30 minutes by week 12 and to forty-five minutes by week 24 as per the ID group. Participants will be phoned fortnightly to provide encouragement and to allow feedback on progress. Only the duration and number of steps of each walking session will be monitored and, therefore, an adequate estimation of total energy expended will not be performed. However, HB participants will be encouraged to regularly increase intensity by walking faster or walking on routes with some degree of incline. Usual care participants will be requested to maintain their usual daily activities and will be reminded of the importance of this regularly throughout the study.

Once randomised, participants will undergo a series of tests including physical function and arterial stiffness measurements at baseline, three months, and six months. All participants will be instructed to refrain from physical activity for 24 hours and food and caffeine intake for three hours prior to each testing session.

### Primary objectives and primary outcome measures

The primary objectives are to compare the effects of supervised intradialytic and home-based exercise training on physical function and arterial stiffness. The primary outcome measures will be outlined below. The hypothesis is that supervised training will be superior to home-based training because the patients will be more likely to meet physical activity guidelines when supervised.

### Physical function

Physical function will be assessed by the 6-Minute Walk Distance (6 MWD) test. This will be performed along a 25-metre stretch of walkway in a quiet hospital corridor. Prior to the test, all participants will undergo a short warm up and familiarisation with the walkway. Participants will be instructed to walk as far as they can during the six minutes. Participants will be allowed to stop and rest if needed and will receive regular encouragement from the investigator throughout the test. Participants, but not investigators, will be blinded to all previous test results.

The secondary outcome measure for physical function is the Timed-Up & Go (TUG) test. The time taken for participants to stand from a seated position, walk 3-metres, turn, and walk back to a chair, and return to the seated position as quickly as possible, is recorded. The better of two attempts will be recorded.

### Arterial stiffness

All measures of arterial stiffness will be taken in the hour preceding dialysis. Aortic (carotid-femoral) pulse wave velocity (PWV) is the primary outcome measure for arterial stiffness. The method involves electrocardiogram-gated sequential applanation tonometry (SPT-301 Mikro-Tip, Millar Instruments, Houston, Texas) of the common carotid and femoral (groin) arteries using the foot-to-foot method (SphygmoCor 7.01, AtCor, Sydney, Australia) as previously described [19].

Additional secondary outcome measures include peripheral (carotid-radial) PWV, AIx and central blood pressures. Peripheral PWV will be assessed as described above from waveforms acquired at the common carotid artery and radial artery. Central blood pressures and AIx will be estimated by pulse wave analysis (PWA) involving radial applanation tonometry performed on the non-fistula arm using customised software (SphygmorCor 7.01, AtCor, Sydney, Australia) with a validated [20] and reproducible [21] generalized transfer function. In patients with a fistula in both arms, the brachial artery (medial to biceps) of the dominant arm will be used. The AIx is defined as the difference between the 2^nd ^and 1^st ^central systolic peaks expressed as a percentage of central pulse pressure[22] Since AIx is influenced by heart rate, the SphygmoCor software also incorporates an algorithm which adjusts AIx to a standard heart rate of 75 beats per minute (bpm) [23]. The central waveform will be calibrated by the average of two measures of automated brachial blood pressure by sphygmomanometry (UA-767, A&D, Saitama, Japan) after five minutes of supine rest.

### Physical activity and self-reported health

Additional secondary outcome measures include physical activity and self-reported health. Items from the Active Australia Questionnaire [24] will be used to quantify the physical activity performed by participants as previously described [25]. Briefly, participants in the exercise groups will be requested to include any training performed in the previous week as part of the study in their response to the questionnaire. The survey measures frequency, intensity and duration of incidental and/or intentional physical activity in the week prior to the time of testing. The total time spent in each activity will be multiplied by an intensity value (3.5 [light], 4 [moderate] and 7 [vigorous]) and used to calculate participants' weekly physical activity in MET.min^-1^.

Self-reported health will be evaluated using the Medical Outcomes Short-Form 36-items (SF-36) Health Survey [26]. Widely used previously in HD populations [27,28] it includes eight independent scales and assesses physical and mental dimensions of health.

### Baseline data

**Figure 1:**
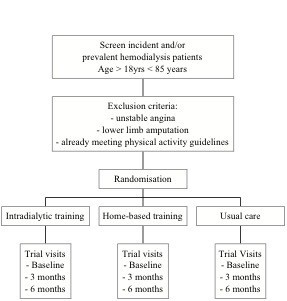
**Flow chart of trial**.

The study flow is summarized in Figure 1 and the study evaluations are outlined in Table 1. After obtaining informed consent patients will be booked two appointments for baseline trial measures. The first of these will occur on a dialysis day. At this visit, additional data will be obtained from the medical records (medical history, medications) and measures of height, weight and blood pressure will be recorded. PWV and PWA measures will then be made 30 minutes prior to dialysis. On the following day subjects will return and the TUG test will be performed followed by the 6 MWD. At the completion of these tests, the investigator will explain and ask the subject to take home two questionnaires (SF 36 and items from Active Australia). Subjects will be asked to complete these surveys and bring them back the next day – at their next dialysis visit. At their next dialysis visit patients will be informed which group they have been randomized to and provided more information about the intervention (or given a brochure if assigned to usual care). Data obtained at the study visits will be transcribed onto case record forms for entry into a specifically designed trial database.

**Table 1:** Outcome measures at baseline, 6 and 12 months

Primary Outcome Measures
Physical Function – six minute walk distance (6 MWD) test

Arterial stiffness – aortic pulse wave velocity (PWV)

Secondary Outcome Measures

Physical Function – timed up and go (TUG) test

Arterial stiffness – peripheral pulse wave velocity

Arterial stiffness – augmentation index (AIx)

Peripheral and Central Blood Pressures

Self reported health – SF 36

Physical Activity – items from the Active Australia questionnaire

### Visits two and three

These visits will occur at approximately three and six months after baseline. Measures and the sequence of these measures will be identical to the baseline visit.

### Adherence to exercise training

Adherence to exercise protocols will be assessed by auditing training diaries. The supervisors will complete the intradialytic participants' training diaries at each training session. Home-based participants will complete diaries themselves and return them at the testing sessions.

### Withdrawal from study

Subjects will be withdrawn from the study at their request, without prejudice, as documented and explained at the time of consenting. Patients who withdraw will be invited to consent to follow-up testing for the remainder of the trial to enable an intention to treat data analysis.

### Sample size calculation

Data from other studies indicates that 6 MWD has the highest variability of the two primary outcome measures (mean ± SD; PWV = 11.1 ± 3.2 [29], 6 MWD = 347 ± 147 m[30]). We assume that this will also mean that changes in 6 MWD will be more variable than changes in PWV. We then assume that a 10% improvement in 6 MWD will be clinically significant. Therefore, with alpha = 0.05, and beta = (1-0.1 = 0.9) we require 17 subjects per group. Allowing for a 20% withdrawal rate we aim to recruit 20 patients per group.

### Statistical analyses

All statistical analyses will be performed using STATA statistical software (STATA 10; Statistical data analysis; Stata Corp; College Station; Texas, USA). Baseline and descriptive data between the groups will be analysed with one-way ANOVA. Binary categorical data will be analysed for variability between the groups using logistic regression. Parametric longitudinal data analysed via two-factor (group, time) analysis of variance (ANOVA) with repeated measures on time using general linear modeling. Weekly physical activity (MET.min^-1^) will be analysed via general linear modelling. Where statistically significant differences are identified, post hoc testing will be performed using Holms tests to locate the means that are significantly different. Statistical significance will be set at p < 0.05.

## Discussion and conclusion

The objective of this trial is to compare a supervised intradialytic training program with a home-based exercise program and usual care. The results of this study will help determine the efficacy of home-based exercise training in hemodialysis patients which may be a more cost effective way of conducting an exercise program as many dialysis units are too crowded and busy to allow for intradialytic exercise programs. This may help in the development of exercise guidelines for dialysis patients. The study will also assess whether the various exercise interventions impact on arterial stiffness a surrogate marker of cardiovascular morbidity in these patients.

## Competing interests

The authors declare that they have no competing interests.

## Authors' contributions

KPK, RGF, JSC and ADW are responsible for the design of this trial, and the construction of the protocol. JES provided advice on the arterial stiffness measures. All authors contributed to, and approved the final manuscript.

## Pre-publication history

The pre-publication history for this paper can be accessed here:

http://www.biomedcentral.com/1471-2369/10/2/prepub
